# Novel Avian Influenza A (H7N9) Virus Induces Impaired Interferon Responses in Human Dendritic Cells

**DOI:** 10.1371/journal.pone.0096350

**Published:** 2014-05-07

**Authors:** Veera Arilahti, Sanna M. Mäkelä, Janne Tynell, Ilkka Julkunen, Pamela Österlund

**Affiliations:** 1 Virology Unit, National Institute for Health and Welfare, Helsinki, Finland; 2 Department of Virology, University of Turku, Turku, Finland; University of Georgia, United States of America

## Abstract

In March 2013 a new avian influenza A(H7N9) virus emerged in China and infected humans with a case fatality rate of over 30%. Like the highly pathogenic H5N1 virus, H7N9 virus is causing severe respiratory distress syndrome in most patients. Based on genetic analysis this avian influenza A virus shows to some extent adaptation to mammalian host. In the present study, we analyzed the activation of innate immune responses by this novel H7N9 influenza A virus and compared these responses to those induced by the avian H5N1 and seasonal H3N2 viruses in human monocyte-derived dendritic cells (moDCs). We observed that in H7N9 virus-infected cells, interferon (IFN) responses were weak although the virus replicated as well as the H5N1 and H3N2 viruses in moDCs. H7N9 virus-induced expression of pro-inflammatory cytokines remained at a significantly lower level as compared to H5N1 virus-induced “cytokine storm” seen in human moDCs. However, the H7N9 virus was extremely sensitive to the antiviral effects of IFN-α and IFN-β in pretreated cells. Our data indicates that different highly pathogenic avian viruses may show considerable differences in their ability to induce host antiviral responses in human primary cell models such as moDCs. The unexpected appearance of the novel H7N9 virus clearly emphasizes the importance of the global influenza surveillance system. It is, however, equally important to systematically characterize in normal human cells the replication capacity of the new viruses and their ability to induce and respond to natural antiviral substances such as IFNs.

## Introduction

In spring 2013 a novel avian influenza A (H7N9) virus emerged in eastern China and by March 2014 has caused two epidemic cluster with nearly 400 reported infections with a mortality of more that 30% [1]. Although many avian influenza viruses circulate endemically in poultry and occasionally cause sporadic infections in humans that are in close contact with live birds at markets or farms, H7N9 virus strain has never before found in humans. Usually, avian influenza virus infections in humans, other than the highly pathogenic H5N1 infections, pose mostly mild or asymptomatic clinical manifestation. However, the novel H7N9 virus appears to be an exception, since it appears to be low pathogenic in birds but highly pathogenic in humans. Most H7N9 virus-infected patients developed severe pneumonia and acute respiratory distress syndrome (ARDS) [2], [3]. It is of great concern if the virus would acquire the ability for sustained human-to-human transmission. Therefore, further characterization of the specific properties of this novel virus such as the molecular determinants of pathogenesis, factors contributing to the ability to replicate in mammalian host, virus – host cell interactions and sensitivity to antiviral substances are of great importance.

Initial genetic analyses revealed that the new H7N9 virus is a reassortant virus with genes originating from at least three different ancestor viruses [3], [4]. More recent evolutionary analyses indicate several separate reassortation events of which the latest one resulted in a shift of the internal genes from an H9N2 virus in domestic birds in China suggesting the poultry and possibly the live poultry markets as the source of human infections [5], [6]. Genetic analyses of the virus revealed possible mutations connected to human adaptation, and the functional analyses confirmed, that unlike other avian viruses, this virus can bind to receptors found in both the upper and the lower parts of the human airways [7]–[10]. This means that the H7N9 virus has an advantage over the other avian strains since it can efficiently grow in a mammalian host, as has been reported in experimental studies with ferrets, mice and human lung tissues [11]–[14]. However, a direct airborne transmission between ferrets has not definitely been demonstrated [15], [16], and neither has the virus so far shown the ability for sustained transmission in humans [17].

The mechanism behind the adverse pathological responses in patients infected with the highly pathogenic avian influenza (HPAI) H5N1 virus is suggested to be hypercytokinemia, “cytokine storm”, which leads to an overwhelming inflammatory response and destruction of the inflamed lung tissues [18]. Most H7N9 virus-infected patients suffered from ARDS and severe lower airway inflammation similar to that seen in patients with H5N1 virus infection. This raises a question whether H7N9 virus can induce a similar immune dysregulation as H5N1 virus, which contributes to the devastating fatal outcome of the disease. Unlike most other inflammatory cytokines, interferons (IFN) mediate antiviral responses in influenza infection by efficiently inhibiting virus replication and growth. We have previously shown that seasonal influenza A viruses induce relatively slow IFN response in human cells [19], [20]. In contrast, influenza B virus induced a much faster IFN response than influenza A virus suggesting a more sensitive recognition of influenza B virus molecules by the infected host cells possibly contributing to a more effective clearance of influenza B infection and milder clinical outcome of the infection [21]. Moreover, we have shown that influenza B virus can trigger early IFN responses directly by the incoming viral particles before any virus replication, transcription or protein synthesis is initiated [21]. This suggests that the early viral entry mechanisms could differ between influenza virus types leading to differences in the activation of antiviral responses.

Since 1997, when the first human cases of highly pathogenic avian H5N1 influenza virus infections were identified, the world has been on alert for potentially new pandemic viruses. The appearance of another dangerous avian virus, namely the H7N9 virus has even enhanced these concerns. To understand the specific properties of this virus strain and the mechanism of the pathogenesis in humans, it is important to study the behavior of H7N9 and H5N1 viruses especially in the human cell system. Dendritic cells (DCs) together with alveolar macrophages reside beneath the epithelium of the respiratory organs and these cells are thus potential targets for influenza viruses. From the epithelial cells influenza viruses spread to DCs and macrophages which coordinate the development of an effective innate immune response against the virus [22], [23]. DCs are specialized antigen-presenting cells inducing the proliferation and activation of T cells, and by this mechanism they are bridging innate and adaptive immune responses [24]. Human monocyte-derived DCs (moDCs) have been shown to express both α-2,3- and α-2,6-linked sialic acids and thus this cell type can be infected with both human and avian adapted influenza virus [25]. Here we studied the activation of innate immune responses in human primary moDCs infected with the human isolate of the novel H7N9 and highly pathogenic H5N1 viruses in comparison with a seasonal H3N2 virus. Interestingly, the novel H7N9 virus showed a weak ability to induce host innate immune responses, while the responses induced by H5N1 virus were extremely strong. The data suggests that the characteristics of influenza viruses with severe clinical outcome at a cellular level may be very different, even completely the opposite from each other.

## Materials and Methods

### Ethics Statement

Adult human blood was obtained from anonymous healthy blood donors through the Finnish Red Cross Blood Transfusion Service (permission no 29/2013, renewed once a year). The permission to import the avian virus strains for research purposes was obtained from the Finnish Food Safety Authority (no 8634/0527/2012). All experiments using infective H5N1 and H7N9 viruses were performed within the Biosafety Level 3 (BSL-3) laboratory of the National Institute for Health and Welfare. Animal experiments related to this study were approved by the Ethical Committee of National Institute for Health and Welfare (permission KTL 2008-02).

### Viruses and Cells

Human seasonal influenza A/Beijing/353/89 (H3N2) virus was grown in embryonated hens’ eggs as described previously [26]. The avian A/Vietnam/1194/04 (H5N1) and A/Anhui/1/13 (H7N9) viruses were grown in Mardin-Darby canine kidney (MDCK) cells. The hemagglutination titers of the virus stocks were 128, 128 and 256, respectively. The virus titers were determined by standard end point dilution assay on MDCK cells that gave the stock virus titers as 10^6,5^ (H3N2), 10^5,4^ (H5N1) and 10^8,5^ (H7N9) TCID_50_, respectively. The propagation of the avian virus stocks and the infection experiments with these viruses were carried out under biosafety level 3 (BSL-3) conditions.

Monocyte-derived DCs (moDCs) were differentiated from peripheral blood monocytes by following a standard procedure [27]. Briefly, peripheral blood mononuclear cells were fractioned by Ficoll-Paque (Pharmacia Biotech) gradient centrifugation followed by centrifugation on a Percoll gradient (Amersham Biosciences) and lymphocyte depletion with anti-CD3 and anti-CD19 magnetic beads (Dynal). MoDCs were differentiated by culturing monocytes in RPMI medium (Sigma Aldrich) supplemented with 0.6 µg/ml penicillin, 60 µg/ml streptomycin, 2 mM L-glutamine and 20 mM HEPES in the presence of 10% FCS (Integro), 10 ng/ml GM-CSF (BioSource) and 20 ng/ml IL-4 (R&D Systems) for 1 week. In each experiment cells from four donors were used separately for virus infection experiments. A549 human lung epithelial cell line (ATCC CCL185) was cultured in Eagle minimal essential medium (Eagle-MEM) (Sigma Aldrich) supplemented with antibiotics, L-glutamine, HEPES, and 10% FCS.

### Infection Experiments

MoDCs and A549 cells were infected with influenza viruses for different times, as indicated in the figures. In the priming experiment moDCs were pretreated with cytokines (see below) for 24 h followed by virus infection for another 24 h. Cells were harvested and samples for qPCR or immunoblotting were prepared. Supernatants for cytokine ELISA measurements were collected before harvesting the cells.

Recombinant human IFN-α2 and IFN-β were purchased from Schering-Plough and TNF-α and IL-1β from Biosource. In cytokine priming experiments moDCs were pretreated with 1, 10 or 100 IU/ml of IFN-α2 or IFN-β, or with 0.5, 5 or 50 ng/ml of TNF-α or with 1, 10 or 100 ng/ml of IL-1β for 24 h before the cells were infected with influenza viruses.

### qPCR

The infected cells from different blood donors were harvested and pooled, and total cellular RNA was isolated using the RNEasy Mini kit (Qiagen) including DNase digestion (RNase-free DNase kit, Qiagen) before transferring the samples out from the BSL-3 facilities. One µg of total cellular RNA was transcribed to cDNA using TaqMan Reverse Transcriptase kit (Applied Biosystems) with random hexamers as primers. cDNAs were amplified by PCR using TaqMan Universal PCR Mastermix and Gene Expression Assays (Applied Biosystems). Influenza A virus-specific primer-probe pair for M1 that detects a highly conserved sequence in M gene of all influenza A viruses was designed by Ward and colleagues and was used with minor modifications [28] The data was normalized to 18S rRNA with TaqMan Endogenous Control kit (Applied Biosystems). Gene expression data is presented as the relative gene expression in relation to the unstimulated samples in order to calculate the fold change achieved by the stimulation.

### Immunoblotting

For protein analyses the cells from different blood donors were pooled, and whole cell lysates were prepared in passive lysis buffer of Dual Luciferase Assay Kit (Promega) containing 1 mM Na_3_VO_4_. Total cellular proteins were denatured in the Laemmli buffer and boiled before transferring the samples out from the BSL-3 facilities. Equal proportion of samples were separated on SDS-PAGE and transferred to Hybond-P polyvinylidene difluoride (PVDF) membranes (Amersham Biosciences). The membranes were blocked with 5% milk protein in PBS (blocking buffer). The rabbit antibodies against IRF1, IRF3, IRF7, MxA, influenza A NP and M1 were prepared as described previously [19], [29]–[31]. The staining was done in blocking buffer at RT for 1 h. Antibodies for phosphorylated IRF3 (P-IRF3), IκBα, and GAPDH were from Cell Signaling Technology, and the staining was done in PBS containing 5% BSA at +4°C overnight. Antibodies against IFITM3 were from Abgent. HRP-conjugated antibodies (Dako) were used in the secondary staining at RT for 1 h. Protein bands were visualized on HyperMax films using an ECL plus system (GE Healthcare).

### ELISA

The secreted levels of multiple subtypes of IFN-α were analyzed from cell culture supernatants using ELISA kit supplied by PBL Biomedical Laboratories. The cytokine levels were analyzed from cell culture supernatants of different blood donors separately.

### Statistical Analysis

Statistical significance of differences between experimental groups was determined through the use of the unpaired, non-parametric Student’s *t* test. Values of *p*<0.05 was considered significant.

## Results

### Replication of Avian-origin H7N9 and H5N1 Viruses in Human moDCs

**Figure 1:**
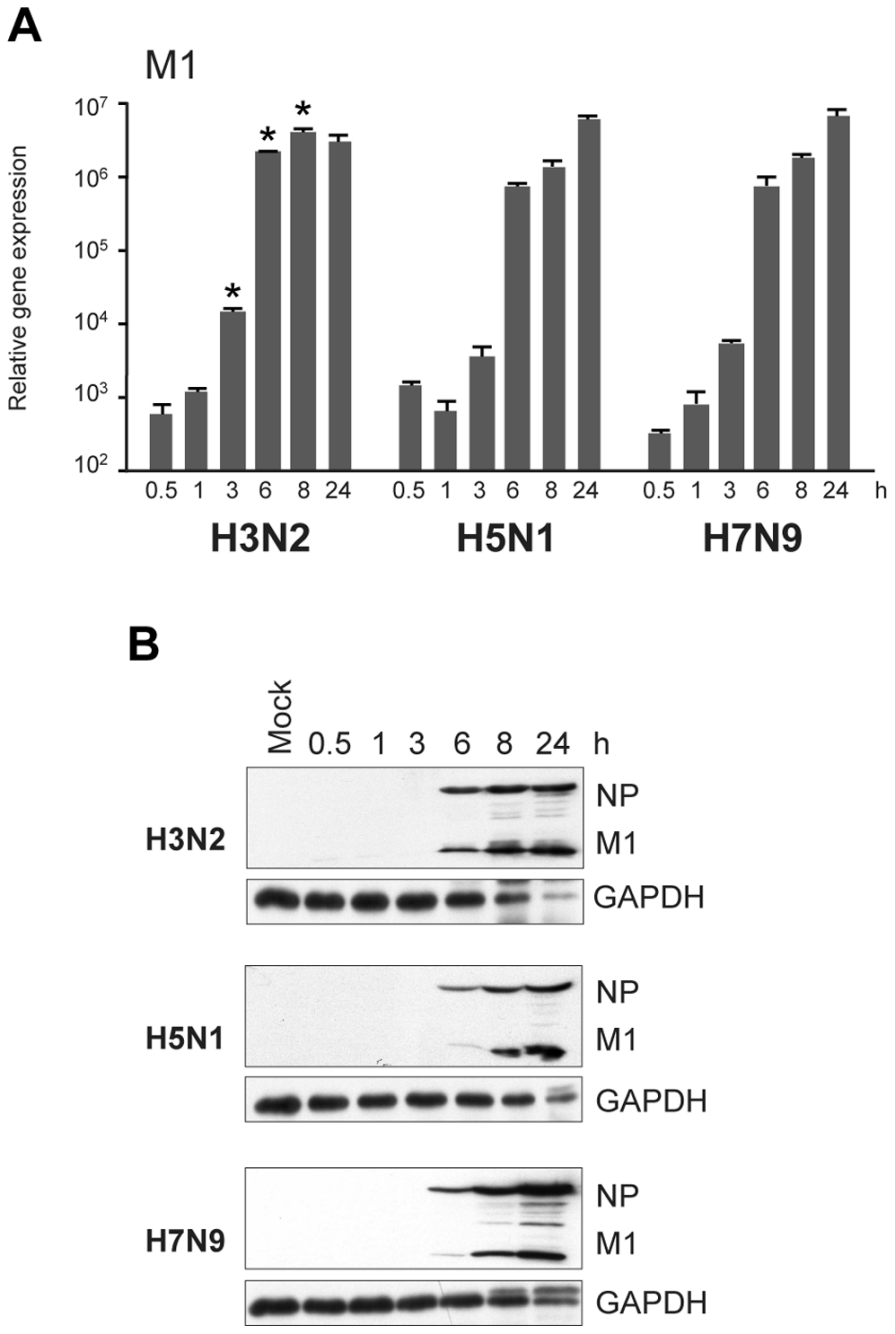
Replication of H3N2, H5N1 and H7N9 viruses in human moDCs. Human primary monocyte-derived DCs (moDCs) were infected with a human seasonal influenza A/Beijing/353/89 (H3N2) virus or avian influenza A/Vietnam/1194/04 (H5N1) or A/Anhui/1/13 (H7N9) viruses at MOI of 1 for various periods of times. **A)** Total cellular RNA was isolated and the expression of influenza M1 RNA was measured by qPCR. The values were normalized to 18S rRNA and presented as relative copy numbers of M1 RNA over the mock sample. The results are presented as means with standard deviation from triplicate measurements. *p<0.05 (Student’s *t* test), difference to both of the other two virus strains. **B)** Whole cell lysates were collected at different time points after infection and viral proteins NP and M1 were analyzed by Western blotting. Results are representative of two experiments both conducted with cells from four different blood donors. For RNA and protein samples the cells from different donors were pooled after the infection experiment.

The severity of influenza A virus infection in humans varies with a fatality rate from 0.1–0.02% of seasonal influenza A viruses to as high as 60% and 30% of the H5N1 and H7N9 viruses, respectively [1]. We analyzed the ability of the novel avian-origin H7N9 virus strain A/Anhui/1/13, HPAI A/Vietnam/1194/04 (H5N1) virus and the seasonal A/Beijing/353/89 (H3N2) virus to replicate and induce immune responses in human primary moDCs. The infectivity of the viruses was determined in MDCK cells with TCID_50_ assay and the titers were 10^8,5^, 10^5,4^ and 10^6,5^, respectively. Quantification of the incoming viral RNA in moDCs after 1 h post infection with serial dilutions of the virus stocks revealed the same relative differences in the infectivity of viral stocks as seen with the TCID_50_ assay (data not shown). This suggests that all studied virus strains were able to infect both cell types with a similar efficacy. Equivalent amounts of infective viruses were used to infect human moDCs. This amount represented a multiplicity of infection (MOI) of 1 for H3N2 virus strain in moDCs that was determined previously [19]. To analyze virus replication in human moDCs, cells were infected with H3N2, H5N1 and H7N9 viruses at doses representing the MOI of 1 for different times and the expression of viral M1 RNA was analyzed by qPCR. The sequence at the target site of the M1 primer-probe pair is fully conserved between influenza A viruses and the sequence homology in this site was 100% between the virus strains used in the present study. All these virus strains were able to replicate efficiently in human moDCs reaching submaximal viral RNA expression after 6 h post infection with comparable RNA levels at the peak time points (Fig. 1A). However, a temporal difference in the replication between human and avian-origin viruses was seen. The human H3N2 virus reached the maximal RNA expression level earlier (at 8 h) than the avian-origin viruses whose RNA expression continued to increase throughout the entire infection period (Fig. 1A). Viral protein expression clearly followed the replication kinetics (Fig. 1B), as analyzed by immunoblotting with antibodies against viral NP and M1 proteins. The avian viruses as well as the H3N2 virus were cytopathic for human cells as evidenced by a detectable decrease in GAPDH protein levels at 24 h after infection (Fig. 1B). The data clearly indicates that avian-origin H5N1 and H7N9 viruses can efficiently replicate in human immune cells.

### Induction of Antiviral IFN Responses in moDCs in Response to Infection with Avian H7N9, H5N1 and Seasonal H3N2 Viruses

**Figure 2:**
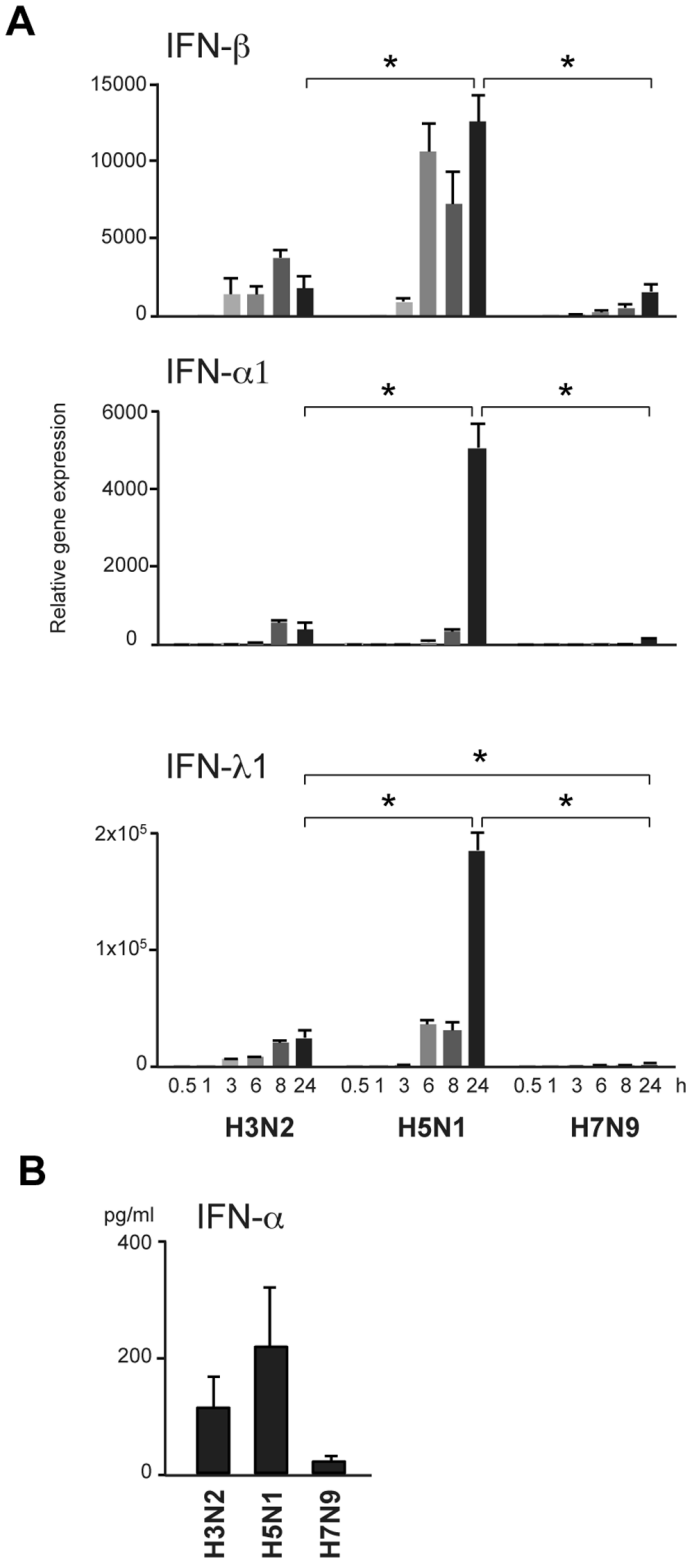
H7N9 virus infection induces weak IFN responses in human moDCs. Human moDCs from four different blood donors were infected separately with influenza A strains A/Beijing/353/89 (H3N2), A/Vietnam/1194/04 (H5N1) and A/Anhui/1/13 (H7N9) at MOI of 1. **A)** Cells were collected at indicated time points after infection and samples from different blood donors were pooled. Total cellular RNA was isolated and prepared for qPCR analyses to detect the expression levels of IFN-β, IFN-α1 and IFN-λ1 genes. The data is representative out of four independent experiments and stand for virus-induced relative expression over a mock sample. The results are presented as means with standard deviations from triplicate measurements. Significant differences between viruses were determined by Student’s *t* test, *p<0.05. **B)** To analyze the secreted IFN-α protein levels cell culture supernatants were collected from virus-infected moDCs at 24 h post infection. The samples were analyzed in duplicate with the ELISA assay. The results are from four different blood donors analyzed separately and presented as mean values with standard deviations.

Antiviral responses against influenza viruses are primarily mediated by IFNs. To analyze the cytokine-mediated antiviral responses we measured IFN gene expression in moDCs infected with seasonal H3N2 or avian-origin H5N1 or H7N9 viruses at MOI of 1 for different periods of times. The quantitation of IFN-β, IFN-α1 and IFN-λ1 mRNA levels revealed that in H7N9 virus-infected cells the antiviral IFN responses were largely impaired and only a weak IFN induction in the latest time points of infection was seen (Fig. 2A). At the same time, we observed remarkably strong IFN gene expression in the cells infected with the HPAI H5N1 virus, whereas the seasonal H3N2 virus induced significantly lower IFN responses. IFN mRNA levels reflected well the secreted protein levels since only a minimal IFN-α production was detectable after 24 h of H7N9 virus infection (Fig. 2B).

**Figure 3:**
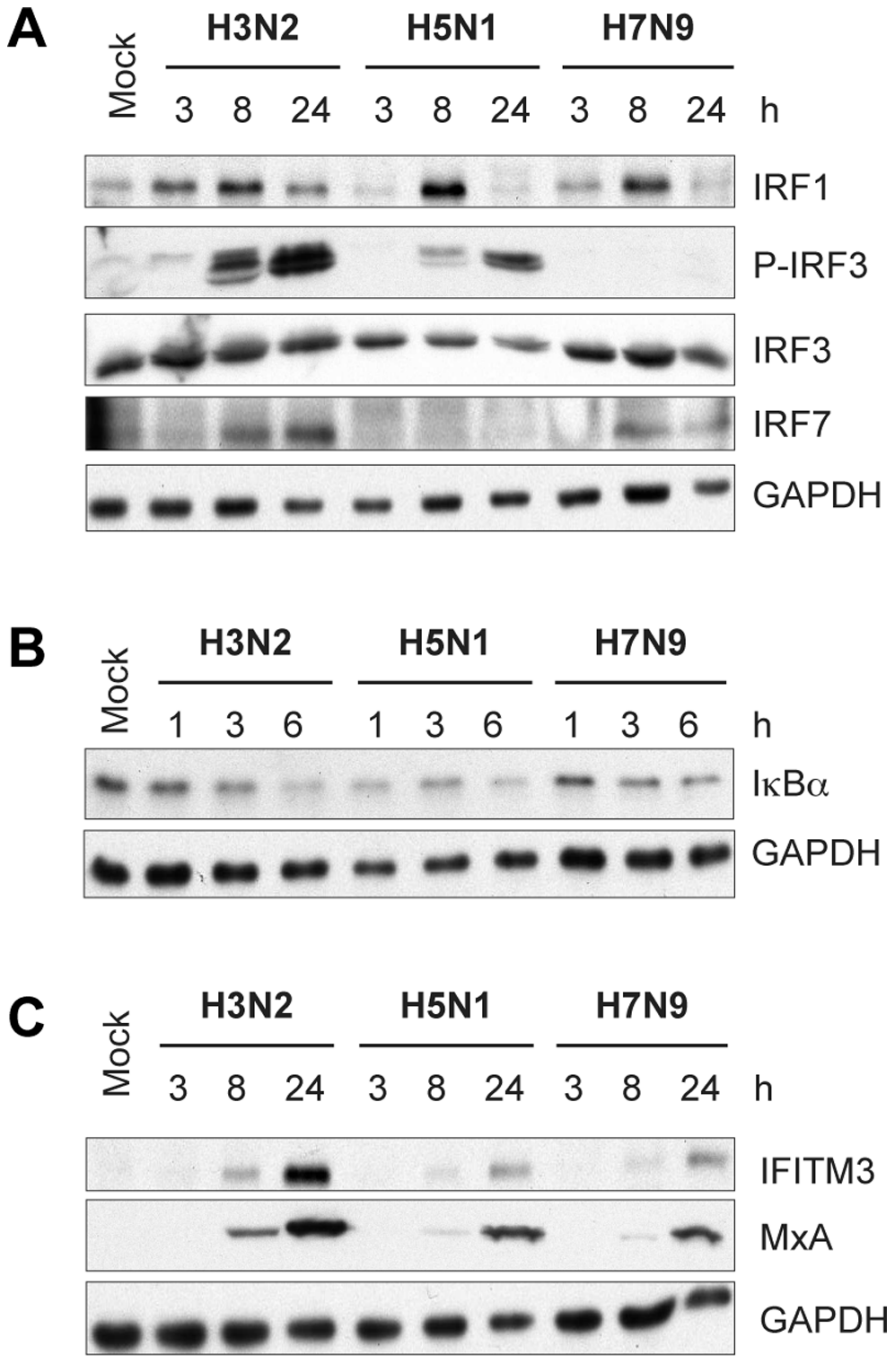
Expression of transcription factors and antiviral proteins in moDCs infected with seasonal or avian influenza viruses. **A)** Immunoblot analysis of IRF1, phosphorylated IRF3 and IRF7 protein levels in moDCs infected with A/Beijing/353/89 (H3N2), A/Vietnam/1194/04 (H5N1) and A/Anhui/1/13 (H7N9) viruses (MOI 1) was carried out from whole cell lysates that were collected at 3, 8, and 24 h time points. **B)** Activation of NFκB detected by visualizing the degradation of its inhibitor IκBα by immunoblot analysis from seasonal and avian influenza virus-infected moDC IκBα expression levels were analyzed in cells collected at 1, 3 and 6 h time points after infection. **C)** Expression of antiviral proteins in influenza virus-infected moDCs were analyzed from total cellular proteins that were separated by SDS-PAGE and immunoblotted with anti-IFITM3 and anti-MxA antibodies. IRF3 and GAPDH were stained for loading control. Cells from four different blood donors were pooled for immunoblot analysis.

Interferon regulatory factor (IRF)-3 is the main transcription factor regulating the expression of antiviral IFN genes, and thus we analyzed the activation of IRF3 by detecting its phosphorylation status. In H7N9 virus-infected moDCs the appearance of phosphorylated form of IRF3 was completely missing (Fig. 3A). However, although the H5N1 virus induced strong IFN responses, the activated form of IRF3 was observed at a lower level as compared to cells infected with the H3N2 virus. In addition to IRF3, the antiviral IFN genes are regulated by several other IRFs [32]. Thus we analyzed the expression levels of IRF1 and IRF7, both of which are inducible by IFNs. We observed that despite the strong IFN expression in H5N1 virus-infected cells, the expression of IRF7 was undetectable in these cells (Fig. 3A). It was also of interest that IRF1 expression, which was strongly induced by all analyzed viruses at the 8 h time point, was completely missing at late times of H5N1 infection. This suggests that H5N1 virus has some mechanism to interfere with the late IFN-mediated antiviral regulation in the infected cells as suggested by earlier reports [33].

Furthermore, NFκB is an important transcription factor regulating inflammatory responses including the proinflammatory cytokine and IFN genes. Thus we analyzed the activation of NFκB by monitoring the degradation of its cytosolic inhibitor IκBα in moDCs infected with seasonal or avian influenza viruses. HPAI H5N1 virus-induced strong and fast degradation of IκBα was visible already at the 1h time point whereas in cells infected with either H3N2 or H7N9 viruses IκBα degradation started to be detectable at 3 h after infection (Fig. 3B). Interestingly in H7N9 virus-infected cells all transcription factor systems measured here, with the exception of IRF3 that was totally missing, were activated almost equally well as in H3N2 virus-infected cells (Fig. 3A and B).

IFNs induce the expression of a large set of antiviral interferon-stimulated genes (ISGs) whose products contribute to the antiviral state in host cells by restricting virus replication. To analyze the antiviral state in H3N2, H5N1 and H7N9 virus-infected human moDCs, the expression of IFN-induced transmembrane protein-3 (IFITM3) was analyzed. This protein is known to restrict replication of multiple viruses that enter the host cytoplasm by an envelope-dependent membrane fusion in endosomes [34], [35]. Moreover, genetic defects in this protein both in knock-out mice and in humans with certain genotype (rs12252-C/C) have been associated with severe pdmH1N1 or H7N9 virus-induced disease [36], [37]. Interestingly, H5N1 and H7N9 virus-induced IFITM3 levels remained extremely low as compared to the levels seen in human H3N2 virus-infected moDCs (Fig. 3C). We also analyzed the expression of another IFN inducible antiviral gene, human myxovirus resistance protein 1 (MxA). In spite of the relatively weak IFN responses in H7N9 virus infected cells, the expression of antiviral MxA protein was clearly detectable in moDCs infected with all the three viruses studied (Fig. 3C). This suggests that even low levels of IFNs induced by the novel H7N9 influenza virus are sufficient to induce the expression of antiviral proteins, such as MxA, in human moDCs. However, although H5N1 virus is inducing extremely strong IFN response, it is still able to disrupt IFN signaling leading to impaired antiviral actions [33].

### Cytokine-mediated Immune Responses in H7N9 and H5N1 Virus-infected moDCs

**Figure 4:**
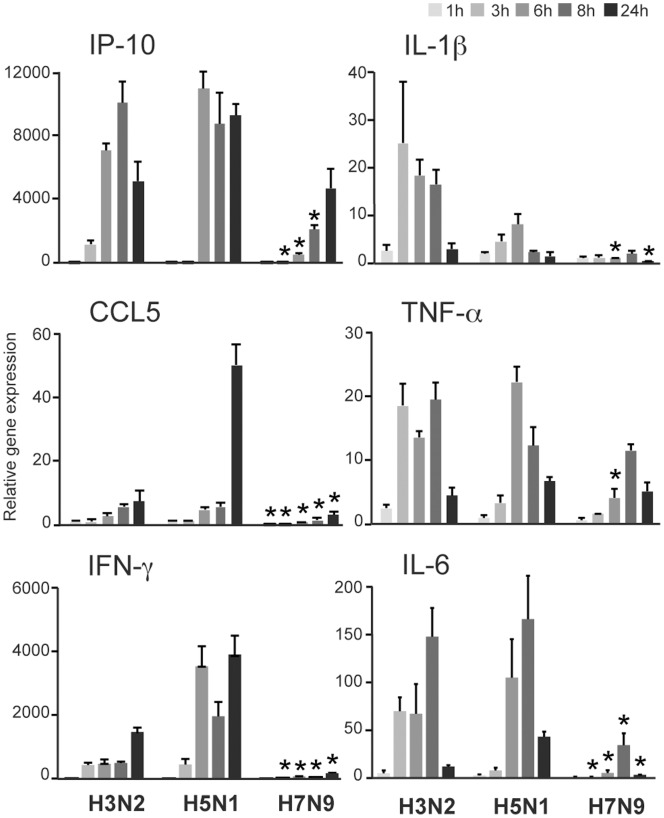
Cytokine expression in moDCs infected with H3N2, H5N1 and H7N9 influenza viruses. Human primary moDCs from four different donors were infected with H3N2, H5N1 or H7N9 viruses (MOI 1) for different times, cells were collected and samples for total cellular RNA were prepared. The expression of pro-inflammatory cytokine and chemokine genes was analyzed by qPCR and the results are presented as fold induction compared to the mock sample. Symbols indicate significant differences between H7N9 and both H3N2 and H5N1 viruses, *p<0.05. The data is representative of two experiments and the results are presented as the mean values with standard deviations of triplicate measurements.

The adverse pathology behind the severe H5N1 infection has been suggested to be partly due to the hypercytokinemia in the site of infection, which eventually leads to tissue destruction and to the development of ARDS [18], [38], [39]. To search for potential immunological mechanisms responsible for severe infections and deaths in H7N9 virus-infected patients, we analyzed the expression profiles of proinflammatory cytokines IFN-γ-induced protein 10 (IP-10), CCL5, IFN-γ, interleukin (IL)-1β, tumor necrosis factor (TNF)-α and IL-6 during the H7N9 infection in human moDCs in comparison with those seen in H5N1 or H3N2 virus-infected cells. Similar to IFN gene expression, other cytokine mRNA levels in H7N9 virus-infected moDCs remained moderate and significantly lower than the levels seen with the H5N1 infection or even with the seasonal H3N2 influenza infection (Fig. 4). Thus, in this simplified cellular model system no signs of cytokine storm could be connected with the novel H7N9 influenza infection.

**Figure 5:**
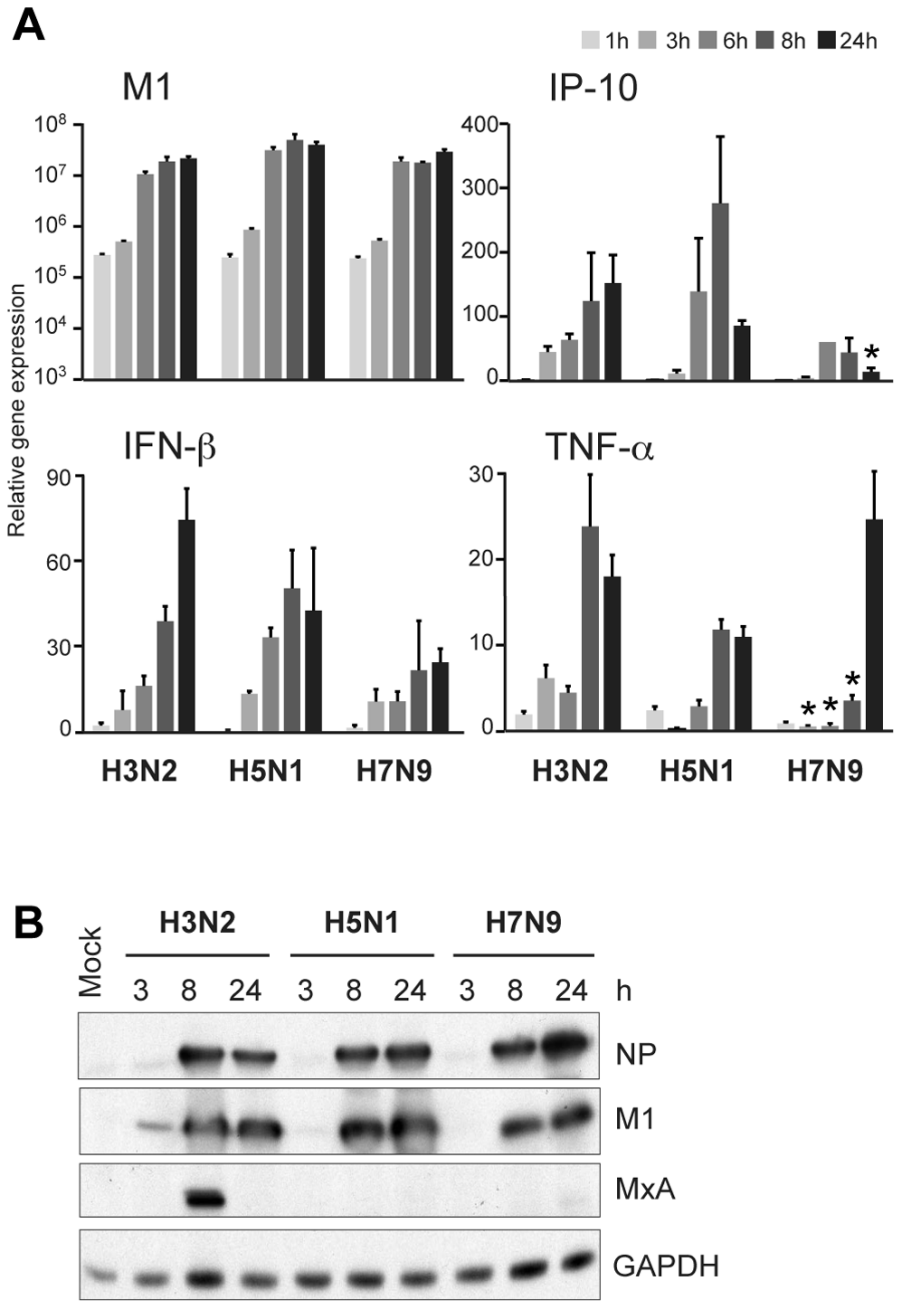
Cytokine responses in A549 lung epithelial cells infected with H3N2, H5N1 and H7N9 influenza viruses. A549 cells were infected with A/Beijing/353/89 (H3N2), A/Vietnam/1194/04 (H5N1) and A/Anhui/1/13 (H7N9) viruses (MOI 5). **A)** Total cellular RNA samples were collected at indicated times for qPCR analyses of viral M1 or cytokine gene expression. The results are presented as fold induction compared to the mock sample. The data is the mean values with standard deviations of triplicate measurements. Statistical differences between H7N9 and both H3N2 and H5N1 viruses were calculated by Student’s *t* test, *p<0.05. **B)** The expression of viral NP and M1 proteins and cellular antiviral protein MxA was visualized by immunoblotting from total cellular lysates collected after 3, 8 and 24 h after infection. GAPDH was stained for loading control.

To confirm these results on impaired cytokine and IFN responses in H7N9 infection, we conducted similar infection experiment in A549 human lung epithelial cells. Also in this cell model all the three influenza viruses replicated efficiently reaching comparable maximal RNA expression levels at late times of infection period (Fig. 5A). Furthermore, the expression levels of IFN-β, IP-10 and TNF-α cytokine genes remained lower than the levels seen in cells infected with H3N2 or H5N1 viruses. Also the antiviral state as measured by the expression of MxA was significantly downregulated by both avian-origin influenza viruses in human A549 lung epithelial cells similar to that seen in human moDCs (Fig. 5B).

**Figure 6:**
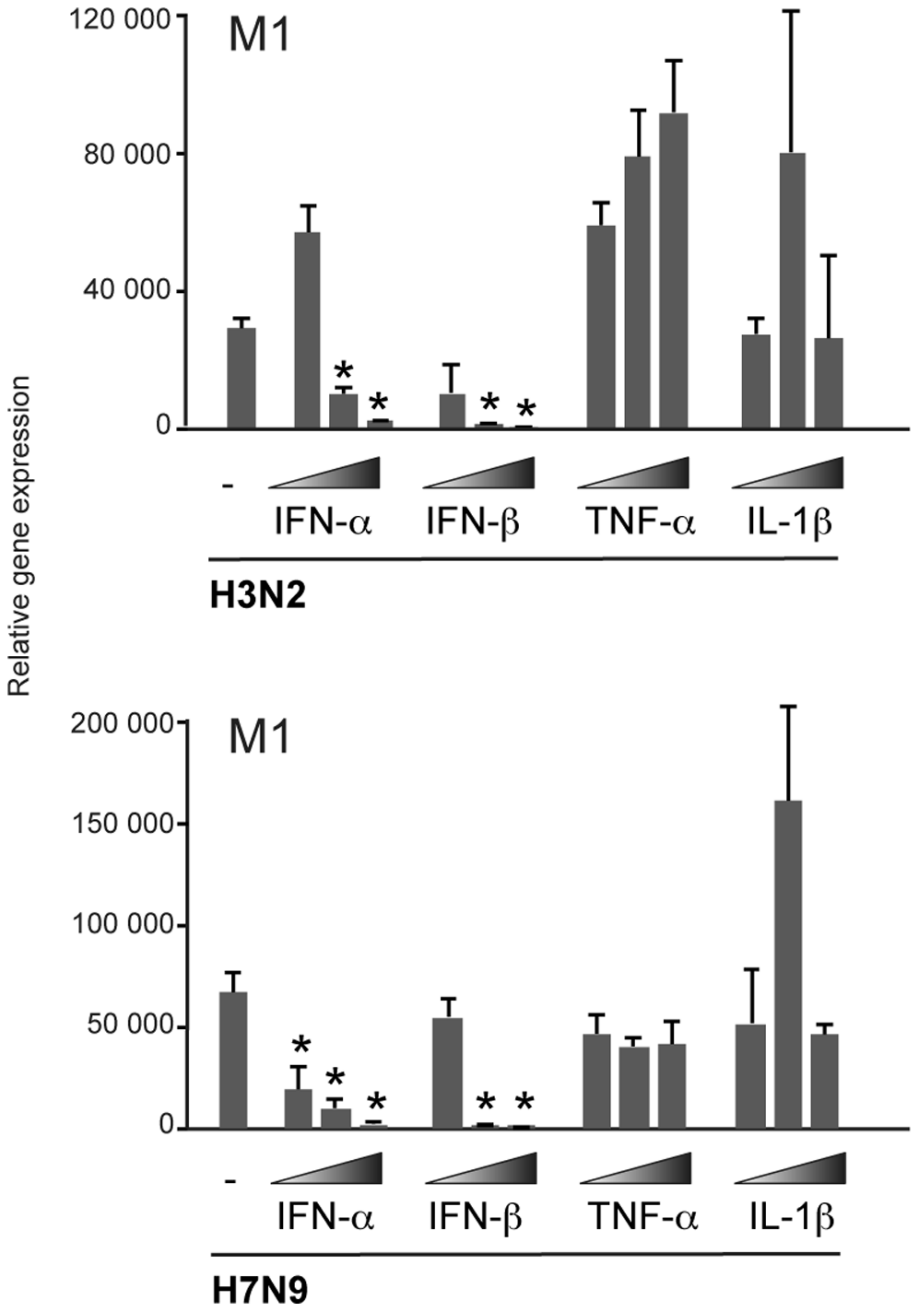
The effects of cytokine priming on the virus replication in moDCs. Monocyte-derived human DCs from four different blood donors were primed with different doses of IFN-α (1, 10 or 100 IU/ml), IFN-β (1, 10 or 100 IU/ml), TNF-α (0.5, 5 or 50 ng/ml), or IL-1β (1, 10 or 100 ng/ml) for 24 h followed by infection with H3N2 or H7N9 viruses for 24 hours. Cells were collected and samples from different donors were pooled for RNA isolation. Virus replication with or without cytokine priming was analyzed by measuring the viral M1 RNA levels by qPCR. The results are presented as relative expression over the mock sample without cytokine stimulation or virus infection. Statistically significant decrease (*p<0.05) from unprimed, infected sample as analyzed with Student’s *t* test. The data is from one experiment and presented as the means with standard deviation of three replicates.

In order to reveal the effects of cytokines produced in response to influenza infection on virus replication, we treated moDCs with type I IFNs or proinflammatory cytokines TNF-α or IL-1β prior to H3N2 or H7N9 virus infection. The analysis of influenza virus M1 gene expression showed clearly that similarly with the earlier reports with H5N1 viruses [40], [41] the H7N9 virus was highly sensitive to the antiviral effects of type I IFNs (Fig. 6). However, the proinflammatory cytokines TNF-α and IL-1β had no decreasing effects on virus replication, suggesting that these cytokines contribute to innate immunity by other mechanisms, like inducing inflammation on the site of infection and recruiting other inflammatory cells to infected tissues.

## Discussion

A new reassortant avian-origin H7N9 influenza virus emerged causing severe infections in humans in spring 2013. Most of the patients with H7N9 infection showed severe influenza-like illness associated with rapidly progressive pneumonia and eventually lower respiratory tract failure [2]. Due to the similarity in the clinical outcome of the H7N9 infection with that of H5N1 infection in humans, we studied whether cytokine responses in H7N9 virus-infected cells resemble the hypercytokinemia associated with the adverse pathology of H5N1 infection [18]. However, to our great surprise, we found that the novel H7N9 virus did not efficiently induce antiviral IFN or proinflammatory cytokine gene expression in human immune cells. The induced levels remained even at a lower level as those seen with a seasonal H3N2 virus infection. The deficiency or lack of activation of the host immune responses in H7N9 infection could contribute to inefficient restriction of the virus spread in inflamed tissues possibly explaining the high viral titers seen in the airway tissues of the H7N9 infected patients [2].

Since IFNs mediate the major host antiviral actions against influenza infection, the deficiency in IFN response in H7N9 virus-infected cells raises a serious concern. Recent reports from patients infected with the novel H7N9 influenza virus [8], [42] are well in line with our observations. In these studies only minimal amounts of IFN-α was detected in the serum specimens of H7N9 virus-infected patients as compared to the specimens of H5N1 virus-infected patients [8]. Moreover, H7N9 virus infection-induced cytokine levels were comparable to those seen in the samples from H3N2 patients or healthy controls [42]. This phenomenon recurred in cultured human alveolar tissue when infected with a human isolate of H7N9 virus, and the H7N9 virus-induced IFN levels remained significantly lower than those induced by avian isolates of H7 subtype viruses [13]. Together these studies support the idea that certain avian viruses such as the HPAI H5N1 infection induces stronger cytokine responses in humans than the partially human-adapted new H7N9 or seasonal viruses. Unlike most avian viruses, the novel H7N9 virus has the ability to bind to the epithelium on both upper and lower human airways [7]–[10]. Together with the deficient IFN responses in H7N9 infection reported in the present study the broad receptor specificity of H7N9 virus could predispose the infected individuals to unlimited virus spread all the way to the lower parts of the lungs leading to ARDS.

In contrast to the hypercytokinemia associated with H5N1 virus-induced pathology, our results show that the H7N9 virus induces only minimal proinflammatory cytokine responses in human cells (Fig. 4 and 5). Very recent study on the whole cell transcriptomic responses to these same three influenza virus infections in human lung epithelial Calu-3 cells was fully consistent with our data. The authors showed that all other influenza viruses except H7N9 significantly upregulated several IFN genes. In addition, the levels for many inflammatory cytokines remained significantly lower in response to H7N9 infection than to infection with H5N1 virus [43]. Also some reports have demonstrated that the cytokine levels in the sera of patients suffering from H7N9 infection remained at lower levels as compared to those seen in H5N1 patients, but yet some increase in proinflammatory cytokine and chemokine levels were seen in the sera of these patients [8]. H7N9 infection was also shown to induce increased cytokine production in the lungs of infected mice [12]. Especially in severe human H7N9 cases, substantial increase in serum IL-6 and IP-10 levels were reported, suggesting a contribution of an excessive inflammatory response to the disease pathology at least in some individuals [42]. However, our studies carried out in cultured human cells are simplified model systems and thus they may not fully reflect the responses seen in serum or organs *in vivo*. Although the clinical outcome in humans in H7N9 and H5N1 virus infections involves ARDS, the mechanism behind the pathology is likely different: the H5N1 virus induces an overwhelming inflammatory response that destroys the lung tissues whereas H7N9 virus can be hypothesized to spread easily in the lungs causing cytopathogenic effects in the absence of IFN-mediated antiviral protection.

The recognition of viral genetic material induces the activation of antiviral signaling pathways, which leads to the activation of the transcription factors, like IRF3, that regulate the expression of antiviral IFN genes. The new H7N9 virus seems to be able to almost completely evade host’s early recognition as evidenced by the lack of IRF3 activation (Fig. 3). On the other hand, H5N1 virus-induced IRF3 phosphorylation remained at a substantially lower level as compared to that seen in H3N2 virus-infected cells (Fig. 3), although IFN gene expression was significantly higher in H5N1 infection (Fig. 2). Altogether this suggests that H7N9 virus is inhibiting (or is unable to activate) IFN responses by blocking the IRF3 activation, whereas H5N1 virus is inducing dysregulation of the IFN genes, presumably by activating multiple transcription factor systems like NFκB (Fig. 3). Influenza viruses have an important means to circumvent the host antiviral activation by its non-structural protein NS1, which is a multifunctional viral protein possessing functions associated with multiple cellular signaling proteins and pathways [44]. The difference in the regulation of IFN responses between these virus strains might be explained by a differential capacity of the viral NS1 protein to suppress the antiviral signaling, as was already suggested by Knepper and coworkers [13]. In this study the authors showed that the H7N9 virus NS1 protein can block IRF3 activation much more effectively than the seasonal H3N2 or avian H7 virus NS1 proteins [13]. However, even if there were differences in the ability of H5N1 and H3N2 virus NS1 proteins to block IRF3 activation in favor of the H5N1 virus NS1 (Fig. 3), this difference could not alone explain the hyperinduction of IFNs seen in H5N1 virus-infected human cells. Instead, studies on H5N1 virus-induced proteomic profiles in human macrophages showed a very early (1 hour post infection) activation of protein synthesis machinery [45]. This could provide a rational explanation for the strong cytokine responses in H5N1 infection, although it cannot be ruled out that NS1 could also have a role in the induction of inflammatory responses. A direct consequence for the expression of IFNs is the induction of ISGs. In spite of the massive IFN induction the expression levels of MxA protein remained relatively low in H5N1 infection (Fig. 3), suggesting that, in addition of being able to induce strong cytokine responses, the H5N1 virus is able to reduce the antiviral state by blocking the expression of ISGs. In fact, it has been shown that the NS1 protein of H5N1 virus disrupts the JAK/STAT-mediated IFN signaling, and this interference is suggested to be mediated by the NFκB-mediated induction of the suppressor of cytokine signaling-1 and 3 (SOCS1/3) [33], [46]. However, if the antiviral state is provoked in advance the H5N1 virus is unable to restrict the antiviral actions [40], [41]. This complexity in interfering with host responses demands further functional and more detailed analyses on the mechanisms underlying the strong inflammatory responses induced by H5N1 virus.

Despite the weak or defective IFN responses in H7N9 infection, the low amount of IFNs produced is still sufficient to induce antiviral state in human cells as evidenced by the expression of MxA protein (Fig. 3). This likely relieves some of the concerns on H7N9 infection lacking antiviral host responses. Supporting this, we demonstrated that H7N9 virus is highly sensitive to the antiviral effects of IFNs (Fig. 6) as has been shown with H5N1 viruses [40], [41]. Genetic analyses of the H7N9 virus NA gene have revealed the R294K mutation known to be associated with resistance to NA inhibitors [4]. However, despite this mutation, analysis of the inhibitory effects of different antiviral compounds against the H7N9 virus has shown that the virus is still susceptible to NA inhibitors as well as to some other antiviral substances [7]. It is noteworthy, that even if the new H7N9 virus acquires resistance to the common antiviral compounds, IFN treatment could be an option for treating the severe, life-threatening forms of H7N9 infections.

The emergence of a new avian influenza A(H7N9) virus in humans revealed that even low pathogenic avian virus strains, by hiding in the poultry and wild birds without any apparent signs for the surveillance efforts, can be a serious threat to human population. Like the H5N1 avian virus, which is highly pathogenic in birds, the novel H7N9 virus causes severe infection and respiratory tract failure in humans [2], [47]. The constant circulation of influenza viruses in the avian reservoir provides a favorable milieu for the development of new reassortant viruses, which have luckily been usually causing only sporadic infections in humans. Here we have shown that H7N9 as well as the H5N1 virus is capable of replicating efficiently in primary human cells without any additional adaptation phases. In addition, we have shown, that the mechanism of pathogenicity between the two avian-origin viruses, H5N1 and H7N9, may be quite different. The deficiency in antiviral IFN responses in H7N9 infection may leave the infected individuals without the host’s own natural antiviral immunity. Although, both viruses are highly pathogenic in humans, no “cytokine storm” was associated with the H7N9 infection. This reinforces the importance of careful diagnosis and antigenic typing for suitable clinical treatment. The lack of activation of host antiviral IFN system could lead to efficient virus spread within the infected host, which further emphasize the pandemic potential of this H7N9 virus.
